# Bioactive Peptide Discovery from Edible Insects for Potential Applications in Human Health and Agriculture

**DOI:** 10.3390/molecules28031233

**Published:** 2023-01-27

**Authors:** Yixian Quah, Shi-Ruo Tong, Joanna Bojarska, Katrin Giller, Sheri-Ann Tan, Zyta Maria Ziora, Tuba Esatbeyoglu, Tsun-Thai Chai

**Affiliations:** 1 Developmental and Reproductive Toxicology Research Group, Korea Institute of Toxicology, Daejeon 34114, Republic of Korea; 2 Department of Physical Science, Faculty of Applied Sciences, Tunku Abdul Rahman University of Management and Technology, Setapak, Kuala Lumpur 53300, Malaysia; 3 Department of Chemistry, Technical University of Lodz, Zeromskiego 116, 90-924 Lodz, Poland; 4 Institute of Agricultural Sciences, ETH Zurich, Universitätstrasse 2, 8092 Zurich, Switzerland; 5 Department of Bioscience, Faculty of Applied Sciences, Tunku Abdul Rahman University of Management and Technology, Setapak, Kuala Lumpur 53300, Malaysia; 6 Institute for Molecular Bioscience, The University of Queensland, Brisbane, QLD 4072, Australia; 7 Department of Food Development and Food Quality, Institute of Food Science and Human Nutrition, Gottfried Wilhelm Leibniz University Hannover, Am Kleinen Felde 30, 30167 Hannover, Germany; 8 Department of Chemical Science, Faculty of Science, Universiti Tunku Abdul Rahman, Jalan Universiti, Bandar Barat, Kampar 31900, Malaysia; 9 Center for Agriculture and Food Research, Universiti Tunku Abdul Rahman, Jalan Universiti, Bandar Barat, Kampar 31900, Malaysia

**Keywords:** antioxidant, antimicrobial, bioactivity, entomophagy, livestock, nutraceutical, peptide purification, protein hydrolysate

## Abstract

In the past decade, there has been fast-growing interest among researchers to discover bioactive peptides from edible insects and to evaluate their potential applications in the management of human, livestock, and plant health. This review summarizes current knowledge of insect-derived peptides and their potential role in tackling human health issues and solving agriculture problems by protecting crops and livestock against their pathogens. Numerous bioactive peptides have been identified from edible insect species, including peptides that were enzymatically liberated from insect proteins and endogenous peptides that occur naturally in insects. The peptides exhibited diverse bioactivities, encompassing antioxidant, anti-angiotensin-converting enzyme, anti-dipeptidyl peptidase-IV, anti-glucosidase, anti-lipase, anti-lipoxygenase, anti-cyclooxygenase, anti-obesity, and hepatoprotective activities. Such findings point to their potential contribution to solving human health problems related to inflammation, free radical damage, diabetes, hypertension, and liver damage, among others. Although most of the experiments were performed in vitro, evidence for the in vivo efficacy of some peptides is emerging. Evidence of the protective effects of insect-derived endogenous antimicrobial peptides in combating farm animal and plant pathogens is available. The ability of insect-derived endogenous neuropeptides to protect plants against herbivorous insects has been demonstrated as well. Nevertheless, the potency of peptides identified from insect protein hydrolysates in modulating livestock and plant health remains a knowledge gap to be filled.

## 1. Introduction

Bioactive peptides may have a positive impact on body functions and thus benefit human health [1,2]. New information reporting their beneficial effects on the health of livestock and plants is also emerging. Bioactive peptides may be produced endogenously in humans, animals, and plants. Furthermore, such peptides can also be released from protein sources by enzymatic hydrolysis or prepared by chemical synthesis [3,4]. While bioactive peptides identified from hydrolyzed food proteins often range between two and twenty amino acid residues, longer endogenous peptides that occur naturally in humans and animals have been discovered [5]. The composition and sequence of amino acids determine the activity of bioactive peptides [6]. Bioactive peptides play important roles in the cardiovascular, immune, nervous, digestive, and endocrine systems. They represent a new generation of bioactive regulators, displaying hormone or drug-like activities, and exhibiting antioxidant, anticancer, antithrombotic, antihypertensive, anti-obesity, anti-inflammatory, opioid, mineral binding, immunomodulatory, antiaging, and antimicrobial effects [7,8,9,10,11]. Bioactive peptides exhibit high specificity in terms of target tissues and consequently possess low or no toxicity. Importantly, they are effective at even relatively low concentrations, which is especially important in the treatment of chronic diseases [5].

The growing interest in bioactive peptides derived from insects has incentivized the scientific community to explore its applications in human health and agriculture due to a burgeoning global population (which is expected to reach 10 billion by 2050), environmental and economic crisis, and rising demand for protein food. In this scenario, entomophagy (consumption of insects) is a future trend in the context of broadly understood sustainability [12,13]. Edible insects are promoted by the Food and Agriculture Organization (Rome, Italy) as a source of food, including feed for animals, preserve agricultural resources, and are used in human and veterinary medicine [14,15]. The European Food Safety Authority (Parma, Italy) established insects as novel foods in 2018 based on the new Regulation 2015/2283 [16] and ruled for the first time in 2021 on the application of novel food from insects, especially from the species *Tenebrio molitor* (mealworm beetle) [17]. Insects belong to the phylum of Arthropods (subphylum Hexapoda)—the largest animal group on Earth. About 2000 species of edible insects belonging to Coleoptera (beetles), Lepidoptera (caterpillars, butterflies, and moths), Hymenoptera (wasps, bees, and ants), Orthoptera (crickets, grasshoppers, and locusts), Hemiptera (cicadas, honey ants, aphids, plant hoppers, leafhoppers, scale insects, and true bugs), Odonata (dragonflies and damselflies), Blattodea (cockroaches and termites), and Diptera (flies), have been reported [12]. Silkworms (*Bombyx mori*), honey bees (*Apis mellifera*), and cochineal (*Dactylopius coccus*) are the most commonly studied [15,18,19,20]. A list of all edible insects was prepared by the University of Wageningen, Netherlands [21]. Edible insects are very popular in Asia, Africa, Oceania, and Latin America. However, Casu marzu and Milbenkäse cheeses, which contain maggots of the cheese fly (*Piophila casei*) or cheese mites, respectively, including their digestive juices, come from Europe (Sardinia and Germany) [22]. It is worth mentioning that insects were valued foods in ancient Greece and Rome [23,24]. The first report on the generation of bioactive peptides from edible insects appeared in 2005 [25]. To date, insect-derived peptides have been reported to exhibit a broad spectrum of bioactivities against inter alia carcinogenesis, immune dysfunctions, and cardiovascular, gastrointestinal, and non-communicable diseases [26,27]. Furthermore, insects are also a rich source of antimicrobial peptides (AMP) [28]. Insect-derived AMPs are effective and non-cytotoxic. They are promising in the fight against bacterial resistance to drugs and the rising emergence of opportunistic pathogens. They can be used as single or synergistic antimicrobial agents, as a replacement for traditional antibiotics, as immunostimulatory agents, or as endotoxin-neutralizing agents [14].

This review presents the recent advances in bioactive peptides derived from edible insects, focusing on their applications in human and livestock health management, as well as the enhancement of plant performance. In this review, we have discussed bioactive peptides derived from 12 species of edible insects. Furthermore, in light of the large numbers of bioactive peptides identified from insect protein hydrolysates, methodologies employed in such studies are summarized. In this review, special attention is given to studies that successfully identified the sequences of bioactive peptides from insects, followed by peptide synthesis and bioactivity validation. Nevertheless, selected examples of bioactive peptide fractions or hydrolysates derived from insect proteins, despite the lack of peptide identification, are briefly discussed where appropriate.

## 2. Purification and Identification of Bioactive Peptides from Insect Protein Hydrolysates

**Figure 1:**
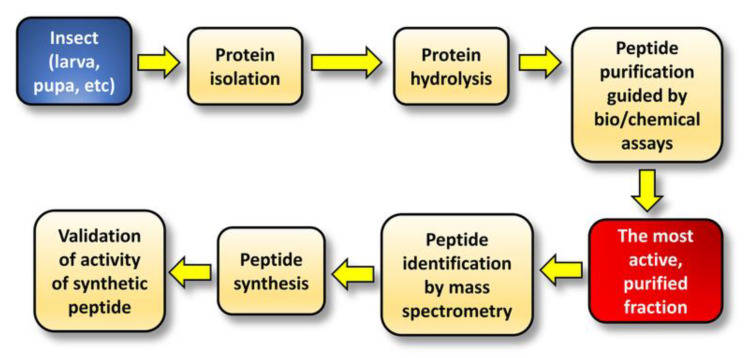
General workflow commonly adopted by researchers in the discovery of bioactive peptides from insect protein hydrolysates.

In the past 10 years, enzymatic hydrolysis has been frequently adopted as a means of producing bioactive peptides from the proteins of edible insects. In such studies, silkworm pupae were relatively popular for the purpose of bioactive peptide discovery [29,30,31,32,33]. Figure 1 shows a general workflow employed by many studies in the discovery of bioactive peptides.

Among the numerous commercially available proteases, alcalase, flavourzyme, and Promod 278P were found to effectively generate functional hydrolysate/peptides from edible insects, leading to the discovery of antioxidant, anti-angiotensin-converting enzyme (ACE), anti-dipeptidyl peptidase-IV (DPP-IV), anti-obesity and hepatoprotective peptides (Table 1). However, alcalase received the most attention as it produced more potent peptides [29,30,34]. This could be because alcalase exhibits both endo- and exo-protease activities, which allows a broad specificity in hydrolysis sites [29], thus providing relatively extensive hydrolysis of the insect proteins. Furthermore, some studies used multiple proteases to generate insect protein hydrolysates, either through sequential hydrolysis with different proteases or in vitro simulation of gastrointestinal digestion in which a combination of multiple gastrointestinal proteases was used. Such use of multiple proteases could improve the degree of hydrolysis and yield more low-molecular-weight peptides when compared to only using a single enzyme in the hydrolysis of insect proteins [31,32,33,35].

Among the studies summarized in Table 1, the proteolysis duration ranged from 80 min to 24 h. The longer the duration of proteolysis, the greater the yield of low-molecular-weight peptides in the resultant hydrolysate. For instance, the 24 h flavourzyme hydrolysate of *Protaetia brevitarsis* contained a much higher level of di- and tri-peptides when compared with other hydrolysates that were produced by a shorter hydrolysis duration [38]. To obtain bioactive peptides from insect proteins, some studies also carried out pre-treatments prior to enzymatic hydrolysis to optimize protein isolation. For example, the proteins of the silkworm and beetle larvae were isolated by using the pH-shift method prior to being used in hydrolysate generation [32,39]. Meanwhile, chemically defatting the edible insects with *n*-hexane and petroleum ether was also performed by some researchers, which could aid in enriching the protein content in the samples [29,32,33,38,39,40].

The isolation of bioactive peptides from the hydrolysate was often accomplished by fractionating the hydrolysate by means of chromatographic and non-chromatographic techniques, guided by in vitro biochemical assays and cell-based bioassays. In the studies presented in Table 1, peptide purification has generally relied on the fractionation of peptides by molecular size. Ultrafiltration was often the first step in peptide purification, probably because of its simplicity and cost-effectiveness. The ultrafiltration membranes used mostly had molecular weight cut-offs (MWCO) of 1, 3, 5, and 10 kDa [30,31,33,34,35,36,37,38]. The peptide fraction with the lowest molecular weight very often showed the highest bioactivity, and thus, was chosen to proceed to the next purification step. The simple peptide sequences in the peptide fraction with the lowest molecular weight might allow a higher exposure of functional groups to bind to target proteins or scavenge free radicals. For example, Wu et al. [32] found that among three ultrafiltration fractions (<5, 5–10, >10 kDa fractions), the <5 kDa fraction exhibited the strongest inhibition of ACE. In studies aiming to discover insect bioactive peptides, a selected ultrafiltration fraction was usually further purified based on molecular sizes by using gel filtration chromatography by the Sephadex G-10, G-15, and/or G-25 resins [31,32,35,38].

Other than the fractionation of peptides by size, the separation of peptides by the charges and hydrophobicity was performed in some investigations. Anion exchange chromatography was frequently used to isolate negatively-charged bioactive peptides, such as GNPWM, an anti-ACE peptide [31], EIAQDFKTDL, an anti-obesity peptide [37], and AGLQFPVGR, a hepatoprotective peptide [36,37]. Phosphate buffers were used as the mobile eluent for the bioactive peptide separation in anion exchange columns; therefore, a desalting step was essential to eliminate the excess salt before the next purification [31,37]. Most studies presented in Table 1 purified the bioactive peptides of interest by using the reversed-phase high-performance liquid chromatography (RP-HPLC) in the last step prior to amino acid sequencing. Hydrophobic amino acids contribute to the activities of anti-ACE, anti-DPP-IV, and antioxidant peptides [30,31,32,33,35,38,39]. Thus, this may rationalize the use of RP-HPLC columns in isolating the aforementioned bioactive peptides from insect protein hydrolysates. However, as exemplified by some studies [38,39], RP-HPLC was not an absolute necessity for purifying peptide fractions for successful peptide identification in the subsequent step.

The peptides present in a purified peptide fraction exhibiting the strongest bioactivity can be identified by means of electrospray ionization-mass spectrometry (ESI-MS), ESI-tandem mass spectrometry (ESI-MS/MS) or Matrix Assisted Laser Desorption/Ionization-MS (MALDI-MS). The robust MS used for peptide identification is often equipped with Time of Flight (TOF), Quadrupole-TOF, Orbitrap, and ion trap. Databases such as UniProt, SwissProt, BIOPEP-UWM, and NCBI were used for peptide identification by searching against the molecular mass-to-charge information from MS or MS/MS [29,30,33,37,39]. Some studies also used in-house or specific databases of edible insects for more specific identification [29,36,37].

The last but most important step is the validation of the bioactivity of the identified peptide sequences. This is invariably carried out by testing the activity of synthetic peptides in chemical or biological assays. Peptides can be synthesized by peptide synthesizer equipment or through a conventional fluorenylmethoxycarbonyl protecting group (FMOC) solid-phase approach or FMOC/*tert*-butyl-based solid-phase protocols [31,37,39]. Usually, the peptides synthesized would be analyzed with HPLC and LC-MS to ensure that the purity of the synthetic peptides ranges from 95 to 99.8% [30,33,34,36,37,39].

After confirming the sequences of the bioactive peptides, some studies also proceeded to perform molecular docking to elucidate the intermolecular interactions between insect-derived bioactive peptides and their target proteins [30,31,32,35,38,39]. The crystal structures of the target proteins that were required for molecular docking simulation were available from the RCSB Protein Data Bank [41]. The three-dimensional (3D) structures of the bioactive peptides were computationally generated using the ChemOffice/ChemDraw software, the Discovery Studio software, or other biopolymer structure preparation tools [30,31,32,38,39]. Collections of 3D structures of dipeptides, tripeptides, and tetrapeptides which consist of all combinations of the 20 natural amino acids, are also freely available in the literature [42]. Such a resource has been used in a previous study when performing molecular docking between peptides of edible insects and target proteins [43]. In a number of studies, molecular docking between bioactive peptides and ACE and DPP-IV was investigated by using the software AutoDock, Discovery Studio, Sulflex-Dock, and an available online tool, Dock 6.9 [31,32,35,38,39]. Besides being used for a further mechanistic understanding of the interactions between bioactive peptides and target proteins, some studies used molecular docking as a means for narrowing down peptide candidates for further experimentation. For example, in the discovery of anti-ACE and antioxidant peptides from the weaver ant protein hydrolysate, the researchers used molecular docking to predict the most promising candidates from a pool of peptides identified by LC-MS/MS, prior to synthesizing only the shortlisted peptides and validating their bioactivities.

## 3. Applications in Human Health Management

**Figure 2:**
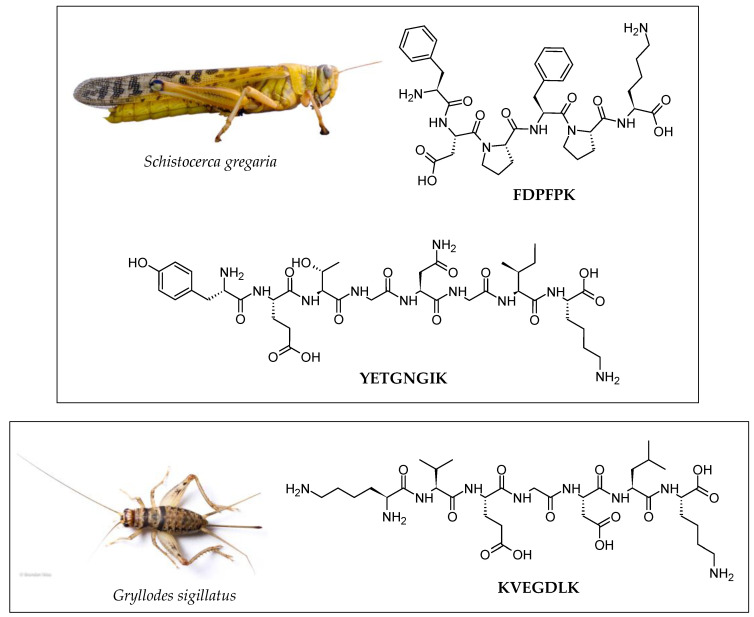
Examples of insect-derived peptides exhibiting antioxidant and anti-glucosidase activities (FDPFPK) as well as anti-ACE activity (YETGNGIK, KVEGDLK) [47]. The two-dimensional structures of the peptides were drawn by using the ACD/ChemSketch freeware (version 2022.1.0, Advanced Chemistry Development, Inc. (ACD/Labs), Toronto, ON, Canada, www.acdlabs.com (accessed on 16 December 2022)). Image of *Schistocerca gregaria* (locust) reprinted with permission from Amada44 [50]. Image of *Gryllodes sigillatus* (cricket) reprinted with permission from Brandon Woo [51].

A wide variety of pathological conditions, including chronic obstructive pulmonary disease (COPD), diabetes complications, obesity, and cancer, have been linked to oxidative stress [44,45,46]. As a result, the development of agents that reduce oxidative stress has piqued the interest of both academic research and the pharmaceutical industry. Many antioxidant peptides have been isolated from edible insects. Most of the examined edible insects’ antioxidant capacities were investigated primarily using 2,2′-azino-bis(3-ethylbenzothiazoline-6-sulfonic acid) (ABTS) and 2,2-diphenyl-1-picrylhydrazyl (DPPH) assays. The peptide FDPFPK (Figure 2) is one of the most potent antioxidant peptides among those listed in Table 2. This synthetic peptide isolated from baked locusts (*Schistocerca gregaria*) showed strong ABTS^•+^ and DPPH^•^ scavenging capacity with IC_50_ values of 0.08 and 0.35 mg/mL, respectively [47,48]. The antioxidant activity of Egyptian cotton leafworm hydrolysate produced by simulated gastrointestinal digestion (SGD) has been studied more thoroughly using cellular and in vivo antioxidant assays [49]. The in vivo *Caenorhabditis elegans* antioxidant model is regarded as an effective model organism for nutritional evaluation, including bioactive peptides. These nematodes have advantages over other in vivo models due to their short life span and, most notably, their high level of gene conservation relative to humans. Therefore, the antioxidant activity of Egyptian cotton leafworm hydrolysate, as measured by the in vivo *Caenorhabditis elegans* antioxidant model, could be regarded as promising and potentially translatable for human health applications.

Edible insects have shown good prospects as a source of anti-hypertensive peptides. Many research groups have discovered ACE inhibitory peptides from common edible insects, including crickets (*Gryllodes sigillatus*), mealworms (*Tenebrio molitor*), locusts, silkworms, grasshoppers (*Sphenarium purpurascens*) [30,47,57], as well as less popular edible insects such as Asian weaver ant (*Oecophylla smaragdina*) and Mexican katydid (*Pterophylla beltrani*) [35,56]. Hypertension affects approximately 1.28 billion adults globally, with the majority living in low- and middle-income countries, according to WHO [58]. Increased renin and/or ACE activity, as well as elevated angiotensin II levels, have been linked to hypertension [59]. Specifically, renin transforms the liver-made decapeptide angiotensinogen into the inactive peptide angiotensin I (Ang I). The ACE then hydrolyses this peptide to produce the vasoactive octapeptide Ang II, which narrows blood vessels [60]. The C-terminal amino acid sequence of the ACE inhibitory peptides determines their affinity to bind with ACE [61]. Peptides with potent ACE inhibitory effect tend to contain hydrophobic amino acids (e.g., Tyr and Trp), hydrophobic side chains (e.g., Val, Leu, and Ile), and positively charged amino acids (e.g., Arg and Lys) at the C-terminus [61]. Some of the most potent ACE inhibitory peptides identified from edible insects, such as YETGNGIK and KVEGDLK, possess these properties. For instance, YETGNGIK (Figure 2), a locust-derived peptide with amino acids Ile (I) and Lys (K) at the C-terminus, exhibited ACE inhibition with an IC_50_ value of 3.25 μg/mL [47]. KVEGDLK (Figure 2), a cricket-derived peptide with Leu (L) and Lys as C-terminal residues, displayed ACE inhibition with an IC_50_ value of 3.67 μg/mL. [47]. The antihypertensive activity of AVF, another anti-ACE peptide identified from *Spodoptera littoralis*, was demonstrated in spontaneously hypertensive rats [53]. The dipeptide VF that was liberated from AVF through the action of in vivo peptidases was proposed to account for the antihypertensive effects of AVF in rats [53]. Furthermore, VF displayed a higher ACE inhibitory activity than AVF. The authors argued that the additional Ala residue might have made the peptide bulkier and prevented it from binding to the ACE binding site, thus decreasing the antihypertensive activity [53]. On the other hand, an HPLC-purified hydrolysate fraction obtained from mealworms was reported to possess antithrombotic activity [55]. Collectively, these results indicate that the bioactive peptides and hydrolysates isolated from the aforementioned edible insects could aid in blood pressure regulation and the prevention of cardiovascular events in humans.

Since hypertension and diabetes share a metabolic pathway, it is highly probable that a given individual will suffer from both conditions [62]. There are different mechanisms to regulate blood glucose in humans. In the small intestine, α-glucosidase catalyzes the breakdown of dextrin into absorbable monosaccharides, thus playing a key role in the digestion of carbohydrates [63]. Alpha-glucosidase inhibitors regulate postprandial hyperglycemia by impeding carbohydrate digestion. The DPP-IV is involved in regulating blood glucose via the inactivation of the incretin hormones glucagon-like peptide-1 (GLP-1) and gastric inhibitory peptide (GIP) [64]. Inhibition of DPP-IV thus prolongs the half-life of incretin hormones, thereby increasing insulin secretion and decreasing blood glucose [63]. Antidiabetic peptides with α-glucosidase inhibitory activity were identified in crickets, mealworms, and locusts [47]. Hydrolysates with α-amylase inhibitory activity were identified from crickets and Mexican katydid [54,56]. The peptide FDPFPK (Figure 2) showed the strongest α-glucosidase inhibition with an IC_50_ value of 5.95 μg/mL among the other identified peptides from the same study [47]. In contrast to ACE inhibitory peptides, the N-terminal of peptides determines their inhibitory activity against DPP-IV. DPP-IV inhibitory peptides may be more potent if they contain Ala (A), Gly (G), Ile, Leu, Pro (P), Met (M), Glu (E), and Val (V) residues at their N-termini. APPDGGFWEWGD, one of the anti-DPP-IV peptides identified from *Tenebrio molitor*, has more than 50% percent of its residues composed of four of the aforementioned amino acids (e.g., A, G, P, and E), including an A residue at the N-terminus [39].

**Figure 3:**
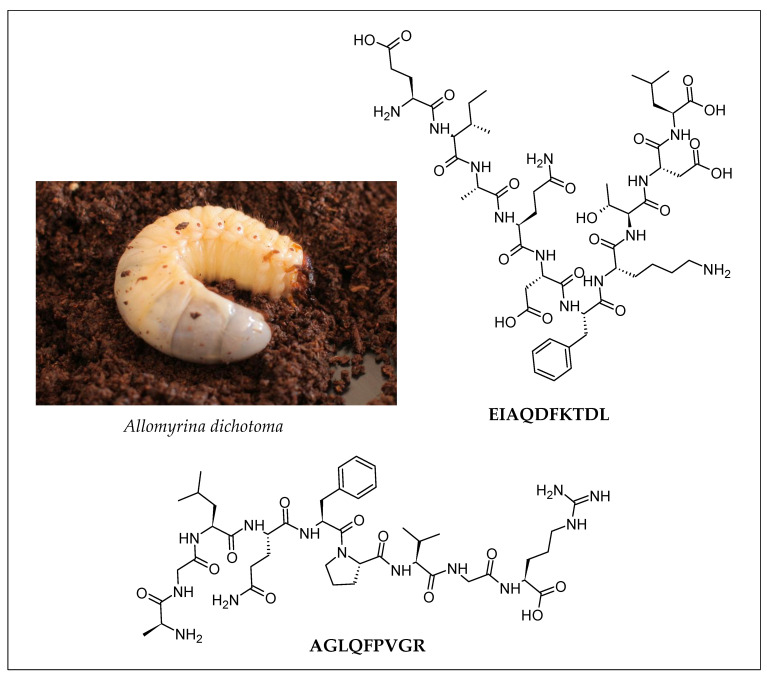
Anti-obesity peptides isolated from the larvae of the Japanese rhinoceros beetle (*Allomyrina dichotoma*). The two-dimensional structures of the peptides were drawn by using the ACD/ChemSketch freeware (version 2022.1.0, Advanced Chemistry Development, Inc. (ACD/Labs), Toronto, ON, Canada, www.acdlabs.com (accessed on 16 December 2022)). Image of *Allomyrina dichotoma* reprinted with permission from ElHeineken [66].

Following hypertension and insulin resistance diabetes, obesity is one of the risk factors for metabolic syndrome. Lipase inhibitors have demonstrated a promising effect on the lipid metabolism of obese individuals. Lipase inhibitors work by preventing fatty acid absorption and thus reducing fatty acid accumulation in the body while also lowering low-density lipoprotein cholesterol levels in the blood and increasing high-density lipoprotein cholesterol levels [65]. Lipase inhibitory peptides from edible insects (e.g., *Gryllodes sigillatus, Tenebrio molitor*, and *Schistocerca gregaria*) were discovered [47]. IIAPPER and AIGVGAIER, lipase-inhibiting peptides isolated from *Gryllodes sigillatus* and *Schistocerca gregaria*, respectively, displayed the strongest inhibitory effect among other peptides in the same study, with an IC_50_ of about 50 μg/mL. An in vivo study discovered an anti-obesity peptide, EIAQDFKTDL (Figure 3), from *Allomyrina dichotoma* Promod 278P hydrolysate. In mice fed a high-fat diet, EIAQDFKTDL significantly improved fatty liver symptoms by decreasing lipid accumulation, catalyzing the breakdown of triglycerides and total cholesterol, lowering blood lipid levels, and lowering body weight [37]. The same research group also established a nonalcoholic fatty liver disease (NAFLD) mouse model that replicates the pathophysiological changes seen in human NAFLD. They used this in vivo model to study the mechanism of action of AGLQFPVGR (Figure 3), a novel anti-obesity peptide isolated from *Allomyrina dichotoma*. AGLQFPVGR acted by activating AMPK/Nrf2 signaling to regulate lipid levels, inhibiting lipid metabolism, and reducing oxidative stress [36]. This peptide also possessed hepatoprotective properties due to its ability to attenuate oxidative stress [36]. These findings reported from in vivo studies are more convincing evidence that peptides isolated from edible insects could be used as a potential treatment for preventing obesity and repairing liver damage in humans.

**Figure 4:**
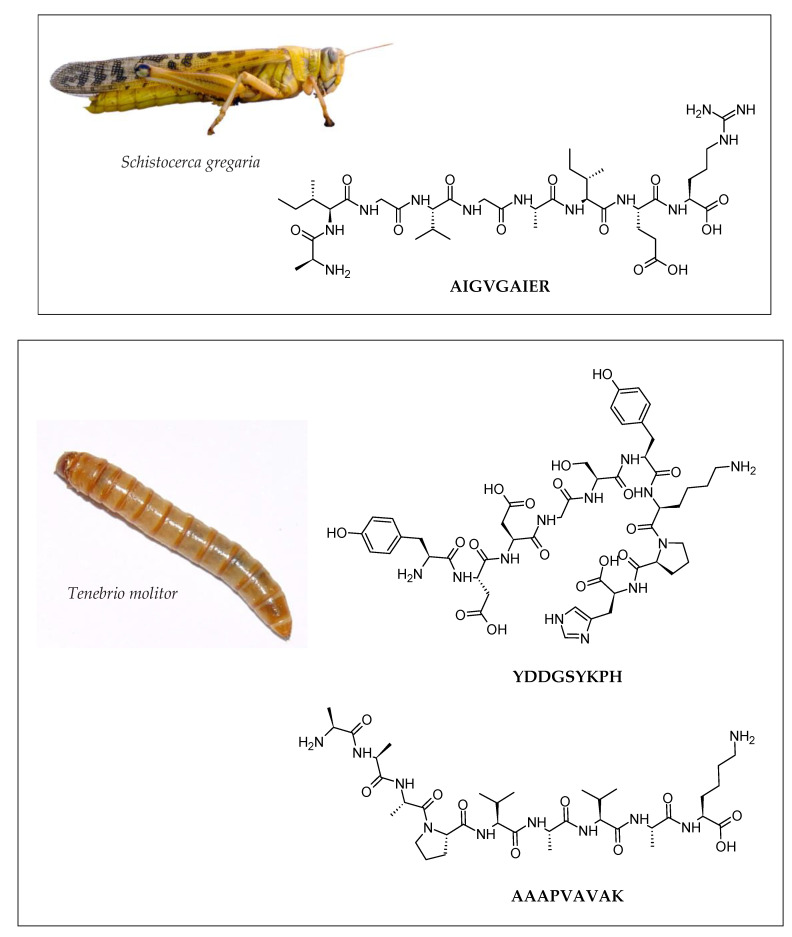
Examples of iron-chelating peptides identified from the locust (*Schistocerca gregaria*) and the larvae of the mealworm (*Tenebrio molitor*) [48]. The two-dimensional structures of the peptides were drawn by using the ACD/ChemSketch freeware (version 2022.1.0, Advanced Chemistry Development, Inc. (ACD/Labs), Toronto, ON, Canada, www.acdlabs.com (accessed on 16 December 2022)). Image of *Schistocerca gregaria* reprinted with permission from Amada44 [50]. Image of *Tenebrio molitor* reprinted with permission from Mnolf [70].

Several metal-chelating peptides were identified from the protein hydrolysates of crickets, mealworms, and locusts [47,48]. Studies suggest that proteolytically hydrolyzed metal chelating peptides are more soluble, easily digested and absorbed, and have distinct physiological activities [67]. Peptides containing Cys (C) and His (H) usually have strong metal chelating activity, as the presence of C and H residues could stabilize Fe^2+^ ions by electron transfer [68]. Intriguingly, none of the peptides tested for Fe^2+^ chelating activity listed in Table 2 contained C residues, and only two peptides, VAPEEHPV and YDDGSYKPH, contained H residues. Comparatively, the strongest Fe^2+^-chelating peptides were AIGVGAIER, YDDGSYKPH, and AAAPVAVAK (Figure 4), which were approximately 190-fold stronger than the positive control, EDTA. In addition, the lipoxygenase (LOX) and cyclooxygenase (COX) inhibitory activities of several isolated peptides from edible insects were investigated. The LOX and COX are key enzymes in the eicosanoid metabolism, thereby modulating inflammatory reactions. Peptides identified from crickets, mealworms, and locusts showed promising anti-inflammatory effects based on their inhibition of LOX and COX [47].

The bioactivities of the aforementioned peptides (Table 2) were extensively demonstrated by using in vitro models. Data on peptide bioactivity in vivo models are gradually emerging [36,37,49,53], although still very limited. Meanwhile, the allergenicity of edible insect-derived bioactive peptides was rarely assessed. The allergenicity of cricket protein hydrolysates has been studied by Hall and colleagues [69]. Compared to the unhydrolyzed cricket proteins, the protein hydrolysates had a lower allergenic effect, as indicated by decreased Immunoglobulin E reactivity to the hydrolysates. There were very few toxicology evaluations on peptides derived from edible insects. In silico analysis predicted the antidiabetic peptides LPDQWDWR and APPDGGFWEWGD [39] and an antithrombotic peptide fraction [55] derived from mealworms to be non-toxic. Meanwhile, the antidiabetic and antihypertension peptide fractions derived from cricket protein hydrolysates were found to have no cytotoxicity against mammalian cells [54]. Overall, numerous insect-derived peptides have shown bioactivities that may improve human health by regulating one or more of the following mechanisms: anti-inflammation, oxidative stress reduction, blood pressure regulation, and blood glucose regulation.

## 4. Applications in Farm Animal Health Management

The beneficial bioactive properties of insect-derived peptides observed in humans and model organisms may also mediate advantageous health effects in livestock. In this context, the potential application of bioactive peptides with antimicrobial properties as alternatives to antibiotics has raised increasing interest [71,72]. In farm animals, the utilization of antibiotics is essential for animal health and welfare as well as food security [73]. The increasing application of antibiotics in livestock production systems represents, however, a major concern regarding the development of antimicrobial resistance that threatens both animal and human health and life [74,75]. Therefore, the importance of reducing the utilization of antibiotics in livestock production systems has been acknowledged in the Sustainable Development Goals [76].

Despite the available data on the antimicrobial effects of insect-derived bioactive peptides, studies in livestock mainly investigated the effects of bioactive peptides derived from sources other than insects [71,72]. Beneficial effects of AMPs from various sources on health and performance have been shown, e.g., in poultry [77,78] and pigs [79,80]. In general, the AMPs from insects comprise around 50 amino acids and are usually cationic [81,82]. The first AMP derived from insects is cecropin, which was isolated from the pupae of the giant silkmoth (*Hyalophora cecropia*) in 1980 [83]. To date, the only study that has investigated the effects of an insect-derived bioactive peptide on livestock physiology applied cecropin AD [84]. Even though the original peptides cecropin A and cecropin D were isolated from insect material (*Hyalophora cecropia*), the cecropin AD gene was expressed in *Bacillus subtilis*, cleaved and purified to obtain the chimeric target peptide (cecropin A(1–11)-D(12–37); KWKLFKKIEKV-GQRVRDAVISAGPAVATVAQATALAK) that was used for dietary supplementation. The cecropin AD was fed to 21-day-old, weaned piglets for 19 days at a 400 mg/kg diet. In comparison to the combination of two antibiotics (kitasamycin (100 mg/kg) and colistin sulfate (800 mg/kg)), no beneficial effects on growth performance were observed when supplementing cecropin AD. In contrast, after challenging the piglets with a single oral dose of 5 mL broth containing 10^9^ colony-forming units/mL *Escherichia coli* K88, cecropin AD improved weight gain and feed efficiency along with reduced diarrhea incidence comparable to the antibiotic treatment. In addition, cecropin AD enhanced the ileal mucosa morphology and modulated the intestinal microbiota composition of the piglets. Different from the antibiotic combination, cecropin AD did not only increase the jejunal secretory immunoglobulins but also increased immunoglobulins and interleukins in serum, indicating a systemic immunomodulating effect of the peptide in weaned piglets. In soybean-meal fed turbot, dietary addition of cecropin AD from the previously described *Bacillus subtilis* expression system also positively modulated intestinal health and prevented the development of enteritis symptoms by shifting the intestinal microbiota towards an anti-inflammatory phenotype [85].

A modified cecropin AD amidated by adding Asn (N) to its C-terminus was expressed in genetically engineered yeast (*Pichia pastoris*), and the culture supernatant was fed to 14-day-old broilers for 28 days at 4 mL/kg [86]. Compared to the control group, the modified cecropin AD increased feed intake, average daily gain, and, consequently, terminal body weight and improved nutrient utilization. Similar to the effects observed in piglets, the intestinal morphology of broilers was improved by cecropin AD. The antimicrobial activity of cecropin is based on its ability to cover the bacterial membrane surface in a carpet-like manner [87]. Cecropin is thus most effective at high concentrations. By causing membrane dissolution, cecropin increases the permeability of the membrane or even completely destroys it [88]. It is assumed that water-soluble short-chain peptides present in many insects could be readily absorbed in animals’ intestines, resulting in systemic effects such as those observed in cecropin AD-fed piglets [84,89].

Insect meals potentially rich in bioactive peptides represent an alternative protein source for the nutrition of monogastric livestock species such as fish, poultry, and pigs [90]. One of the most used insects is the black soldier fly (BSF, *Hermetia illucens*), including its larvae, which possess antimicrobial effects by reducing *Escherichia coli* O157:H7 and *Salmonella* ser. Enteritidis in chicken manure [91]. Even though the active compounds were not identified in that study, peptides and other small molecules extracted from BSF larvae exhibited a broad-spectrum antimicrobial activity against a range of Gram-positive and Gram-negative bacteria [92]. In addition, AMPs such as defensins and defensin-like peptides have been detected in BSF and might have contributed to the observed effects [93,94]. Defensins mediate their anti-bacterial effects by forming channels in the bacterial membrane, which increases its permeability [95].

Overall, insect-derived bioactive peptides with antimicrobial properties represent an interesting alternative to the utilization of antibiotics in livestock production. Nevertheless, Chernysh and colleagues [96] reported a rapid increase in bacterial resistance toward individually tested AMPs. In contrast, when utilizing an insect AMP complex consisting of defensins, cecropins, diptericin, and proline-rich peptides, no signs of antimicrobial resistance were detected. It is thus suggested that combining AMPs with different modes of action may reduce the risk of the development of resistant bacteria.

In addition to the antimicrobial properties of insect-derived peptides, bioactive peptides with antioxidant properties are also of interest to livestock nutrition. The continuous exposure of livestock animals to pathogenic microbes results in the activation of immune cells and, consequently, the production of reactive oxygen species. Excessive formation of reactive oxygen species can promote inflammatory processes and impair animal health and productivity. In this context, BSF protein hydrolysates were shown to possess antioxidant properties when compared to fish meal and chicken meal, potentially providing advantageous effects on animal health [97]. These results were obtained in vitro and thus require confirmation in vivo. Still, the antioxidant activity of insect proteins has been demonstrated upon ingestion by fish and chicken [98,99], suggesting that antioxidant peptides might be involved in mediating the observed effect.

## 5. Applications in Plant Health Management

Evidence in the literature substantiates the efficacy of endogenous AMPs of insect origin in protecting against plant diseases. Plant diseases due to microbes and insects have led to an approximately 30% reduction in the yield of staple crops worldwide, with agricultural losses amounting to billions of dollars [100]. In view of the above, the effects of endogenous AMPs, specifically those from edible insects, are discussed below.

In addition to the antimicrobial effects of cecropin observed in animals, as presented in the previous section, cecropin was also found to be functional against both phytopathogenic bacteria and fungi [101]. Other AMPs which can target plant pathogens with different modes of action, such as sarcotoxin, attacins, defensins, and metchnikowin, were isolated from various insects. However, these AMPs displayed broad-spectrum activities against more than one bacterium/fungus. AMPs with specific inhibition towards a single pathogen are more desirable [102].

The creation of transgenic plants expressing insect AMPs to resist bacterial and fungal infections is widely implemented in the agricultural sector. The gene coding for the apidaecins, AMPs from honeybees, was genetically engineered into the genome of the potato plant. The transgenic potato demonstrated resistance to infections from plant pathogens of the *Erwinia* genus and *Agrobacterium* species [103]. In addition, a tomato plant expressing cecropin was shown to resist wilt and spot diseases caused by the pathogenic bacteria *Ralstonia solanacearum* and *Xanthomonas campestris*, respectively [104]. The fusion of two or more AMPs to form chimeric AMPs prior to transformation into plants was reported to increase the potency of the recombinant peptides to overcome future infections [105,106]. Nevertheless, the production of foreign AMP genes within the host could interfere with the plant’s own gene expression system, which may indirectly affect the plant’s physiology and fitness [82].

There are also insect-derived neuropeptides that could confer plants with insecticidal properties against herbivorous insects. These neuropeptides generally modulate the insect’s behavior and physiology by interfering with the arthropod’s developmental processes, which include reproduction, energy metabolism, and growth [107]. The injection of the neuropeptide Allatostatin Manse-AST from the tobacco hornworm (*Manduca sexta*) into the larvae of the tomato moth (*Lacanobia oleracea*) led to reduced feeding, growth retardation, and a higher mortality rate of up to 80% [108]. This neuropeptide is made up of the sequence pEVRFRQCYFNPISCF-OH [109]; it can inhibit foregut peristalsis of insect larvae, producing the aforementioned insecticidal effects [108].

The Australian funnel web spider (*Hadronyche versuta*) produces a neurotoxin known as ω-Atracotoxin (ACTX)-Hv1a (Hvt), which acts as a potent insect-specific calcium channel blocker [110,111,112]. Structurally, this 37-amino acid peptide possesses a disulfide-bonded globular hydrophobic core with a protruding finger-like β- hairpin. The core comprises side-chains of Ile 5, Cys 11, Cys 17, Cys 22, and Cys 36 [111]. For stability, this inhibitory peptide has a cystine knot motif, a signature among neurotoxin peptides [110,111]. This peptide was expressed recombinantly in *Escherichia coli*, purified, and tested topically on the caterpillars of the cotton bollworm (*Helicoverpa armigera*) and Egyptian cotton leafworm (*Spodoptera littoralis*). It was found that the LD_50_ of this toxin was as low as 2 to 4 pmol for both species. Furthermore, no larvae survived the ordeal after 24 h of exposure [112]. The same research team proceeded to create a transgenic tobacco plant harboring the foreign Hvt gene. It was revealed that the mutated plant successfully expressed the recombinant peptide, thus, protecting it against attack from the larvae of *Helicoverpa armigera* and *Spodoptera littoralis* [112]. The detached leaf toxicity assay clearly indicated that the leaves of transgenic tobacco were not as badly damaged by the insects compared to the wild-type leaves. Other homologous neuropeptides of ω-ACTX-Hv1a, such as ω-ACTX-Ar1a from Sydney funnel-web spider (*Atrax robustus*), also exerted similar biopesticidal properties but with lower efficacy [110].

## 6. Future Perspectives and Conclusions

Although the bio-evaluation of insect-derived peptides has been intensively advancing during the last decade, there is still a great need for more research. For example, the effectiveness of insect-derived antioxidant peptides was mostly demonstrated through free radical scavenging assays. In the future, it would be worth investigating whether such peptides can also target cellular sources of reactive oxygen species (ROS), not only the ROS themselves. One example of ROS-producing enzymes is the nicotinamide adenine dinucleotide phosphate oxidase (NOX) family, which is associated with a number of pathological events [113]. The potency of insect-derived antioxidant peptides as NOX inhibitors could be evaluated in future research. Peptides that target ROS generation mechanisms might improve human health more effectively than peptides that simply scavenge ROS. In this review, it is apparent that structural differences among the bioactive peptides outnumber their structural similarities. This could be attributed at least partly to diversity in the protein sequences in different insect samples and/or the use of different proteases among the studies. The structure–activity relationship (SAR) of the bioactive peptides has not been thoroughly established. Facilitated by an increasing number of peptide sequences identified from insects with time, future focus on SAR should be intensified. This could contribute to a deeper understanding of the structural factors underlying the bioactivities of the peptides and may contribute towards future designs of more potent bioactive peptides. On the other hand, a more allergenicity risk assessment should be performed to increase the public’s confidence in insect-derived peptides. For potential therapeutic peptide candidates with potent bioactivities, in vitro and in vivo toxicity testing is strongly recommended.

The activities of insect-derived peptides are still scarcely explored in livestock species. It can be assumed, though, that the bioactive effects of such peptides vary between ruminant and monogastric species due to their differing digestive processes. The peptides might not resist microbial degradation during rumen passage in ruminants but might instead exert antimicrobial effects on the rumen microbiota and thus modulate fermentation processes. Future studies should investigate a potential targeted application, specifically on antimicrobial peptides from insects as livestock nutrition. In addition, the concentrations and activities of bioactive peptides in insect meals should be investigated to evaluate their supply to livestock via the basal diet. A maintained activity of bioactive peptides in processed insect meals would circumvent the resource-costly extraction of bioactive peptides when provided with intact insect meals. Confirming the beneficial health effects of bioactive peptides in meals may further promote the application of those products as livestock feed. Regarding the currently low availability of insect products and their comparably high costs, large-scale production in the case of a desired targeted supplementation of livestock species with insect-derived bioactive peptides could be a further challenge. 

The effectiveness of insect protein hydrolysates and peptides derived from the latter as plant biostimulants is still a knowledge gap. Interestingly, plant-derived protein hydrolysates have been shown to improve the germination, yield, and quality of various crops, such as tomatoes and lettuces, by modulating plant microbiomes as well as alleviating environmental stresses [114,115]. Hence, more attention should be given to this area of research in order to further exploit the agricultural value of edible insect hydrolysates.

To summarize, current evidence in the literature supports the potential application of edible insect-derived peptides in the treatment of human diseases and other health-related issues. Many such peptides were purified and identified from enzymatic hydrolysates of insect proteins. By contrast, the potential application of edible insect-derived peptides in tackling livestock and plant health issues is still poorly investigated. In light of the future trend of entomophagy, the research on health-promoting peptides from edible insects is anticipated to attract increasing attention worldwide, including a further focus on the agricultural application of insect-derived peptides.

## Figures and Tables

**Table 1:** Examples of purification and identification methodologies used in the discovery of bioactive peptides from insect hydrolysates.

Insect	Peptide Sequence (Validated Activity)	Enzymatic Hydrolysis	Peptide Purification Strategy	Peptide Identification	Reference
Larva of the Japanese rhinoceros beetle (*Allomyrina dichotoma*)	EIAQDFKTDL (Anti-obesity) AGLQFPVGR (Hepatoprotective)	Promod 278P *, pepsin, trypsin, protease NP, pancreatin, alphalase NP, alkaline protease, alcalase, neutrase, protamex	UF IEC RP-HPLC	MS/MS analysis	[36,37]
Larva of the white-spotted flower chafer (*Protaetia brevitarsis*)	SY, PF, YPY, WI (Anti-ACE)	Flavourzyme	UF GFC	LC-MS/MS	[38]
Mealworm (*Tenebrio molitor*)	LPDQWDWR, APPDGGFWEWGD (Anti-DPP-IV)	Flavourzyme *, alcalase, papain, trypsin	GFC	LC-MS/MS	[39]
Mealworm (*Tenebrio molitor*)	LE, AKKHKE (Hepatoprotective)	Alcalase *, flavourzyme, neutrase	UF Solid-phase extraction RP-HPLC	LC-MS LC-MS/MS	[34]
Asian weaver ant larva and pupa mixture (*Oecophylla smaragdina*)	FFGT, LSRVP (Anti-ACE) CTKKHKPNC (Antioxidant)	SGD (Pepsin and trypsin)	UF GFC RP-HPLC	LC-MS/MS	[35]
Silkworm pupa (*Bombyx mori*)	AAEYPA, AKPGVY (Antioxidant)	Alcalase *, papain, trypsin	UF RP-HPLC	LC-MS/MS	[30]
Silkworm pupa (*Bombyx mori*)	SWFVTPF, NDVLEF (Antioxidant)	Alcalase *, Prolyve, Flavourzyme, Brewers Clarex	RP-HPLC	LC-MS/MS	[29]
Silkworm pupa (*Bombyx mori*)	FKGPACA, SVLGTGC (Antioxidant)	Acidic protease, followed by neutral protease	UF GFC RP-HPLC	LC-MS/MS	[33]
Silkworm pupa (*Bombyx mori*)	ASL (Anti-ACE)	SGD (pepsin, trypsin, and α-chymotrypsin)	UF GFC RP-HPLC	LC-MS	[32]
Silkworm pupa (*Bombyx mori*)	GNPWM (Anti-ACE)	Neutral protease	UF IEC GFC RP-HPLC	MALDI-MS/MS	[31]

* Enzyme generating the most active hydrolysate, which was selected for peptide purification. ACE: Angiotensin-I-converting enzyme, DPP-IV: Dipeptidyl peptidase-IV, GFC: Gel filtration chromatography, IEC: Ion exchange chromatography, LC: Liquid chromatography, MALDI: Matrix Assisted Laser Desorption/Ionization, MS: Mass spectrometry, MS-MS: Tandem mass spectrometry, RP-HPLC: Reversed-phase High-Performance Liquid Chromatography, SGD: Simulated gastrointestinal digestion, UF: Ultrafiltration.

**Table 2:** Edible insects used for the generation of bioactive peptides and their potential applications in human health management.

Insect	Peptide/Hydrolysate	Bioactivity *	Potential Application	References
Cricket (*Gryllodes sigillatus*)	IIAPPER	ACE inhibition: IC_50_, 6.93 μg/mL Lipase inhibition: IC_50_, 49.44 μg/mL α-Glucosidase inhibition: IC_50_, 22.86 μg/mL Radical scavenging activity (ABTS assay): IC_50_, 15.62 mg/mL Antioxidant activity (DPPH assay): IC_50_, 1.01 mg/mL Fe^2+^ chelating activity: IC_50_, 0.14 mg/mL LOX inhibition: IC_50_, 8.21 mg/mL COX inhibition: IC_50_, 8.16 mg/mL	Anti-hypertension, antidiabetic, weight control, antioxidant, and anti-inflammation	[47,48]
	LAPSTIK	ACE inhibition: IC_50_, 11.14 μg/mL Lipase inhibition: IC_50_, 104.95 μg/mL α-Glucosidase inhibition: IC_50_, 45.60 μg/mL Radical scavenging activity (ABTS assay): IC_50_, 15.69 mg/mL Antioxidant activity (DPPH assay): IC_50_, 0.66 mg/mL Fe^2+^ chelating activity: IC_50_, 0.456 mg/mL LOX inhibition: IC_50_, 12.3 mg/mL COX inhibition: IC_50_, 8.39 mg/mL		
	VAPEEHPV	ACE inhibition: IC_50_, 18.85 μg/mL Lipase inhibition: IC_50_, 100.13 μg/mL Radical scavenging activity (ABTS assay): IC_50_, 3.49 mg/mL Antioxidant activity (DPPH assay): IC_50_, 0.29 mg/mL Fe^2+^ chelating activity: IC_50_, 0.155 mg/mL LOX inhibition: IC_50_, 7.56 mg/mL COX inhibition: IC_50_, 8.61 mg/mL		
	KVEGDLK	ACE inhibition: IC_50_, 3.67 μg/mL Lipase inhibition: IC_50_, 115.44 μg/mL α-glucosidase inhibition: IC_50_, 18.37 μg/mL Radical scavenging activity (ABTS assay): IC_50_, 2.88 mg/mL Antioxidant activity (DPPH assay): IC_50_, 8.73 mg/mL Fe^2+^ chelating activity: IC_50_, 0.122 mg/mL LOX inhibition: IC_50_, 10.08 mg/mL COX inhibition: IC_50_, 8.43 mg/mL		
Mealworm (*Tenebrio molitor*)	NYVADGLG	ACE inhibition: IC_50_, 12.09 μg/mL Lipase inhibition: IC_50_, 115.59 μg/mL α-Glucosidase inhibition: IC_50_, 20.37 μg/mL Radical scavenging activity (ABTS assay): IC_50_, 3.71 mg/mL Antioxidant activity (DPPH assay): IC_50_, 0.99 mg/mL Fe^2+^ chelating activity: IC_50_, 0.198 mg/mL LOX inhibition: IC_50_, 9.27 mg/mL COX inhibition: IC_50_, 9.75 mg/mL		
	AAAPVAVAK	ACE inhibition: IC_50_, 8.31 μg/mL Lipase inhibition: IC_50_, 57.69 μg/mL α-Glucosidase inhibition: IC_50_, 10.92 μg/mL Radical scavenging activity (ABTS assay): IC_50_, 0.94 mg/mL Antioxidant activity (DPPH assay): IC_50_, 1.02 mg/mL Fe^2+^ chelating activity: IC_50_, 0.108 mg/mL LOX inhibition: IC_50_, 8.36 mg/mL COX inhibition: IC_50_, 9.02 mg/mL		
	YDDGSYKPH	ACE inhibition: IC_50_, 5.81 μg/mL Lipase inhibition: IC_50_, 117.94 μg/mL Radical scavenging activity (ABTS assay): IC_50_, 1.02 mg/mL Antioxidant activity (DPPH assay): IC_50_, 1.91 mg/mL Fe^2+^ chelating activity: IC_50_, 0.107 mg/mL LOX inhibition: IC_50_, 6.49 mg/mL COX inhibition: IC_50_, 8.07 mg/mL		
	AGDDAPR	ACE inhibition: IC_50_, 8.34 μg/mL Lipase inhibition: IC_50_, 77.46 μg/mL α-glucosidase inhibition: IC_50_, 19.47 μg/mL Radical scavenging activity (ABTS assay): IC_50_, 1.89 mg/mL Antioxidant activity (DPPH assay): IC_50_, 1.83 mg/mL LOX inhibition: IC_50_, 7.03 mg/mL COX inhibition: IC_50_, 9.01 mg/mL		
Locust (*Schistocerca gregaria*)	GKDAVIV	ACE inhibition: IC_50_, 12.82 μg/mL Lipase inhibition: IC_50_, 53.17 μg/mL α-Glucosidase inhibition: IC_50_, 15.94 μg/mL Radical scavenging activity (ABTS assay): IC_50_, 1.97 mg/mL Antioxidant activity (DPPH assay): IC_50_, 1.3 mg/mL Fe^2+^ chelating activity: IC_50_, 0.101 mg/mL LOX inhibition: IC_50_, 8.95 mg/mL COX inhibition: IC_50_, 8.91 mg/mL		
	AIGVGAIER	ACE inhibition: IC_50_, 14.4 μg/mL Lipase inhibition: IC_50_, 49.95 μg/mL α-Glucosidase inhibition: IC_50_, 13.04 μg/mL Radical scavenging activity (ABTS assay): IC_50_, 1.28 mg/mL Antioxidant activity (DPPH assay): IC_50_, 0.51 mg/mL Fe^2+^ chelating activity: IC_50_, 0.101 mg/mL LOX inhibition: IC_50_, 20.29 mg/mL COX inhibition: IC_50_, 8.96 mg/mL		
	FDPFPK	ACE inhibition: IC_50_, 79.25 μg/mL Lipase inhibition: IC_50_, 96.75 μg/mL α-Glucosidase inhibition: IC_50_, 5.95 μg/mL Radical scavenging activity (ABTS assay): IC_50_, 0.08 mg/mL Antioxidant activity (DPPH assay): IC_50_, 0.35 mg/mL Fe^2+^ chelating activity: IC_50_, 0.137 mg/mL LOX inhibition: IC_50_, 2.85 mg/mL COX inhibition: IC_50_, 7.40 mg/mL		
	YETGNGIK	ACE inhibition: IC_50_, 3.25 μg/mL Lipase inhibition: IC_50_, 94.91 μg/mL Radical scavenging activity (ABTS assay): IC_50_, 2.96 mg/mL Antioxidant activity (DPPH assay): IC_50_, 1.4 mg/mL Fe^2+^ chelating activity: IC_50_, 0.257 mg/mL LOX inhibition: IC_50_, 7.56 mg/mL COX inhibition: IC_50_, 8.83 mg/mL		
Silkworm pupa (*Bombyx mori*)	AAEYPA	Radical scavenging activity (ABTS assay): IC_50_, 70.32 μg/mL Antioxidant activity (DPPH assay): IC_50_, 70.83 μg/mL	Antioxidant	[30]
	AKPGVY	Radical scavenging activity (ABTS assay): IC_50_, 34.32 μg/mL Antioxidant activity (DPPH assay): IC_50_, 58.50 μg/mL		
Silkworm pupa (*Bombyx mori*)	SWFVTPF NDVLFF	Antioxidant activity in AAPH induced HepG2 cells: 36.96% Antioxidant activity in AAPH induced HepG2 cells: 30.43%	Antioxidant	[29]
Silkworm pupa (*Bombyx mori*)	FKGPACA SVLGTGC	Radical scavenging activity (ABTS assay): IC_50_, 0.312 mM Radical scavenging activity (ABTS assay): IC_50_, 0.181 mM	Antioxidant	[33]
Silkworm pupa (*Bombyx mori*)	ASL	ACE inhibition IC_50_, 102.15 μM	Anti-hypertension	[32]
Silkworm pupa (*Bombyx mori*)	GNPWM WW	ACE inhibition: IC_50_, 21.70 μM ACE inhibition: IC_50_, 10.76 μM	Anti-hypertension	[31]
Silkworm pupa (*Bombyx mori*)	PNPNTN	Promoted Concanavalin A-induced splenocyte proliferation at 100 μg/mL	Immunomodulation	[52]
Asian weaver ant (*Oecophylla smaragdina*)	FFGT LSRVP	ACE inhibition: IC_50_, 19.5 μg/mL ACE inhibition: IC_50_, 52.7 μg/mL	Anti-hypertension	[35]
	CTKKHKPNC	Radical scavenging activity (ABTS assay): IC_50_, 38.4 μg/mL Antioxidant activity (DPPH assay): IC_50_, 48.2 μg/mL	Antioxidant	
Mealworm (*Tenebrio molitor*)	LPDQWDWR APPDGGFWEWGD	DPP-IV inhibition: IC_50_: 0.15 mg/mL DPP-IV inhibition: IC_50_: 1.03 mg/mL	Antidiabetic	[39]
Larva of the Japanese rhinoceros beetle (*Allomyrina dichotoma*)	EIAQDFKTDL	In vivo model: HFD mouse model Reduction in body weight, TG, TC, LDL/VLDL, glucose, ALT, and AST levels. Increased HDL level compared to HFD vehicle control. In vitro model: 3T3-L1 cells Lipid accumulation assay: 30.22% of control (lowest among the identified peptides)	Anti-obesity, weight control	[37]
Larva of the Japanese rhinoceros beetle (*Allomyrina dichotoma*)	AGLQFPVGR	In vivo model: HFD mouse model Inhibited fat deposition in the liver of HFD mouse Restored SOD, GPx, and GR gene expression levels, improving antioxidant capacity of liver cells.	Anti-obesity, weight control, hepatoprotective	[36]
Cotton leafworm (*Spodoptera littoralis*)	VF AVF	In vivo model: SHR rat model A single oral administration of each peptide to SHR significantly reduced blood pressure.	Anti-hypertensive	[53]
Egyptian cotton leafworm (*Spodoptera littoralis*)	SGD hydrolysate	In vivo model: *Caenorhabditis elegans* ORAC: IC_50_, 0.052 mg/mL Radical scavenging activity (ABTS assay): IC_50_, 0.24 mg/mL Cellular antioxidant activity was similar to ascorbic acid (positive control) Protective effect in vivo against acute oxidative stress	Antioxidant	[49]
Cricket (*Gryllodes sigillatus*)	Cationic peptide fraction from sequential alcalase and SGD hydrolysates	ACE inhibition: IC_50_, 1.922 μg/mL α-amylase inhibition: IC_50_, 96.75 μg/mL α-Glucosidase inhibition: IC_50_, 13.902 μg/mL	Antidiabetic and anti-hypertension	[54]
Yellow mealworms (*Tenebrio molitor*)	RP-HPLC fraction of pepsin and trypsin hydrolysate	Antithrombotic activity at 0.2 mg/mL: approximately 30%	Antithrombotic	[55]
Mexican katydid (*Pterophylla beltrani*)	SGD hydrolysate	ACE inhibition: IC_50_, 0.49 mg/mL	Anti-hypertension	[56]
	<3 kDa fraction of SGD hydrolysate	ACE inhibition: IC_50_, 1.44 mg/mL α-amylase inhibition: IC_50_, 0.68 mg/mL	Antidiabetic, Anti-hypertension,	

* All bioactivities were results obtained from in vitro models unless otherwise stated. AAPH: 2,2′-Azobis(2-amidinopropane) dihydrochloride, ABTS: 2,2′-Azino-bis(3-ethylbenzothiazoline-6-sulfonic acid), ACE: Angiotensin-converting enzyme, COX: Cyclooxygenase, DPPH: 2,2-Diphenyl-1-picrylhydrazyl, DPP-IV: Dipeptidyl peptidase IV, GPx: Glutathione peroxidase, GR: Glucocorticoid receptor, HDL: High-density lipoprotein cholesterol, HFD: High-fat diet, LDL: Low-density lipoprotein cholesterol, LOX: Lipoxygenases, ORAC: Oxygen radical antioxidant capacity, SGD: Simulated gastrointestinal digestion, SHR: Spontaneously hypertensive rat, SOD: Superoxide dismutase, TC: Total cholesterol, TG: Triglycerides, VLDL: Very low-density lipoprotein cholesterol.

## Data Availability

Not applicable.

## References

[1] Apostolopoulos V., Bojarska J., Chai T.-T., Feehan J., Kaczmarek K., Matsoukas J.M., Paredes Lopez O., Saviano M., Skwarczynski M., Smith-Carpenter J. (2022). New advances in short peptides: Looking forward. Molecules.

[2] Chai T.-T., Ee K.Y., Kumar D.T., Manan F.A., Wong F.-C. (2020). Plant bioactive peptides: Current status and prospects towards use on human health. Protein Pept. Lett..

[3] Jakubczyk A., Karas M., Rybczynska-Tkaczyk K., Zielinska E., Zielinski D. (2020). Current trends of bioactive peptides—New sources and therapeutic effect. Foods.

[4] Chai T.-T., Law Y.-C., Wong F.-C., Kim S.-K. (2017). Enzyme-assisted discovery of antioxidant peptides from edible marine invertebrates: A review. Mar. Drugs.

[5] Apostolopoulos V., Bojarska J., Chai T.-T., Elnagdy S., Kaczmarek K., Matsoukas J., New R., Parang K., Lopez O.P., Parhiz H. (2021). A global review on short peptides: Frontiers and perspectives. Molecules.

[6] Fields K., Falla T.J., Rodan K., Bush L. (2009). Bioactive peptides: Signaling the future. J. Cosmet. Dermatol..

[7] Akbarian M., Khani A., Eghbalpour S., Uversky V.N. (2022). Bioactive peptides: Synthesis, sources, applications, and proposed mechanisms of action. Int. J. Mol. Sci..

[8] Korhonen H., Pihlanto A. (2006). Bioactive peptides: Production and functionality. Int. Dairy J..

[9] Sánchez A., Vázquez A. (2017). Bioactive peptides: A review. Food Qual. Saf..

[10] Shahidi F., Zhong Y. (2008). Bioactive peptides. J. AOAC Int..

[11] Zaky A.A., Simal-Gandara J., Eun J.B., Shim J.H., Abd El-Aty A.M. (2022). Bioactivities, applications, safety, and health benefits of bioactive peptides from food and by-products: A review. Front. Nutr..

[12] Iriti M., Vitalini S. (2022). Edible insects—A new trend in functional food science. Funct. Food Sci..

[13] Guiné R.P.F., Correia P., Coelho C., Costa C.A. (2021). The role of edible insects to mitigate challenges for sustainability. Open Agric..

[14] Mlcek J., Borkovcova M., Bednarova M. (2014). Biologically active substances of edible insects and their use in agriculture, veterinary and human medicine—A review. J. Cent. Eur. Agric..

[15] Van Huis A. (2013). Potential of insects as food and feed in assuring food security. Annu. Rev. Entomol..

[16] European Parliament and Council of the European Union (2015). Regulation (EU) 2015/2283 of the European Parliament and of the Council of 25 November 2015 on novel foods, amending Regulation (EU) No 1169/2011 of the European Parliament and of the Council and repealing Regulation (EC) No 258/97 of the European Parliam. Off. J. Eur. Union.

[17] EFSA Panel on Nutrition N.F., Allergens F., Turck D., Castenmiller J., De Henauw S., Hirsch-Ernst K.I., Kearney J., Maciuk A., Mangelsdorf I., McArdle H.J. (2021). Safety of dried yellow mealworm (*Tenebrio molitor* larva) as a novel food pursuant to Regulation (EU) 2015/2283. EFSA J..

[18] Bullard B., Linke W.A., Leonard K. (2002). Varieties of elastic protein in invertebrate muscles. J. Muscle Res. Cell Motil..

[19] Sun-Waterhouse D., Waterhouse G.I.N., You L., Zhang J., Liu Y., Ma L., Gao J., Dong Y. (2016). Transforming insect biomass into consumer wellness foods: A review. Food Res. Int..

[20] Nongonierma A.B., FitzGerald R.J. (2017). Unlocking the biological potential of proteins from edible insects through enzymatic hydrolysis: A review. Innov. Food Sci. Emerg. Technol..

[21] Jongema Y. http://www.wur.nl/en/Expertise-Services/Chair-groups/Plant-Sciences/Laboratory-of-Entomology/Edible-insects/Worldwide-species-list.htm.

[22] Halloran A., Vantomme P., Hanboonsong Y., Ekesi S. (2015). Regulating edible insects: The challenge of addressing food security, nature conservation, and the erosion of traditional food culture. Food Secur..

[23] Bergier E. (1941). Peuples Entomophages et Insectes Comestibles: Etude sur les Moeurs de L’homme et de L’insecte.

[24] Bodenheimer F.S. (1951). Insects as Human Food: A Chapter of the Ecology of Man.

[25] Vercruysse L., Smagghe G., Herregods G., Van Camp J. (2005). ACE inhibitory activity in enzymatic hydrolysates of insect protein. J. Agric. Food Chem..

[26] Acosta-Estrada B.A., Reyes A., Rosell C.M., Rodrigo D., Ibarra-Herrera C.C. (2021). Benefits and challenges in the incorporation of insects in food products. Front. Nutr..

[27] Lange K.W., Nakamura Y. (2021). Edible insects as future food: Chances and challenges. J. Future Foods.

[28] Wu Q., Patočka J., Kuča K. (2018). Insect antimicrobial peptides, a mini review. Toxins.

[29] Cermeño M., Bascón C., Amigo-Benavent M., Felix M., FitzGerald R.J. (2022). Identification of peptides from edible silkworm pupae (*Bombyx mori*) protein hydrolysates with antioxidant activity. J. Funct. Foods.

[30] Khammuang S., Sarnthima R., Sanachai K. (2022). Purification and identification of novel antioxidant peptides from silkworm pupae (*Bombyx mori*) protein hydrolysate and molecular docking study. Biocatal. Agric. Biotechnol..

[31] Tao M., Wang C., Liao D., Liu H., Zhao Z., Zhao Z. (2017). Purification, modification and inhibition mechanism of angiotensin I-converting enzyme inhibitory peptide from silkworm pupa (*Bombyx mori*) protein hydrolysate. Process Biochem..

[32] Wu Q., Jia J., Yan H., Du J., Gui Z. (2015). A novel angiotensin-I converting enzyme (ACE) inhibitory peptide from gastrointestinal protease hydrolysate of silkworm pupa (*Bombyx mori*) protein: Biochemical characterization and molecular docking study. Peptides.

[33] Zhang Y., Wang J., Zhu Z., Li X., Sun S., Wang W., Sadiq F.A. (2021). Identification and characterization of two novel antioxidant peptides from silkworm pupae protein hydrolysates. Eur. Food Res. Technol..

[34] Cho H.-R., Lee S.-O. (2020). Novel hepatoprotective peptides derived from protein hydrolysates of mealworm (*Tenebrio molitor*). Food Res. Int..

[35] Pattarayingsakul W., Nilavongse A., Reamtong O., Chittavanich P., Mungsantisuk I., Mathong Y., Prasitwuttisak W., Panbangred W. (2017). Angiotensin-converting enzyme inhibitory and antioxidant peptides from digestion of larvae and pupae of Asian weaver ant, *Oecophylla smaragdina*, Fabricius. J. Sci. Food Agric..

[36] Fan M., Choi Y.-J., Tang Y., Kim J.H., Kim B.-g., Lee B., Bae S.M., Kim E.-K. (2021). AGL9: A novel hepatoprotective peptide from the larvae of edible insects alleviates obesity-induced hepatic inflammation by regulating AMPK/Nrf2 signaling. Foods.

[37] Bae S.M., Fan M., Choi Y.-J., Tang Y., Jeong G., Myung K., Kim B.-g., Kim E.-K. (2020). Exploring the role of a novel peptide from *Allomyrina dichotoma* larvae in ameliorating lipid metabolism in obesity. Int. J. Mol. Sci..

[38] Lee J.H., Kim T.-K., Yong H.I., Cha J.Y., Song K.-M., Lee H.G., Je J.-G., Kang M.-C., Choi Y.-S. (2022). Peptides inhibiting angiotensin-I-converting enzyme: Isolation from flavourzyme hydrolysate of *Protaetia brevitarsis* larva protein and identification. Food Chem..

[39] Tan J., Yang J., Zhou X., Hamdy A.M., Zhang X., Suo H., Zhang Y., Li N., Song J. (2022). *Tenebrio molitor* proteins-derived DPP-4 inhibitory peptides: Preparation, identification, and molecular binding mechanism. Foods.

[40] Kim T.-K., Lee J.-H., Yong H.I., Kang M.-C., Cha J.Y., Chun J.Y., Choi Y.-S. (2022). Effects of Defatting Methods on the Physicochemical Properties of Proteins Extracted from Hermetia illucens Larvae. Foods.

[41] Burley S.K., Bhikadiya C., Bi C., Bittrich S., Chen L., Crichlow G.V., Christie C.H., Dalenberg K., Di Costanzo L., Duarte J.M. (2020). RCSB Protein Data Bank: Powerful new tools for exploring 3D structures of biological macromolecules for basic and applied research and education in fundamental biology, biomedicine, biotechnology, bioengineering and energy sciences. Nucleic Acids Res..

[42] Prasasty V.D., Istyastono E.P. (2019). Data of small peptides in SMILES and three-dimensional formats for virtual screening campaigns. Data Brief.

[43] Wong F.-C., Ong J.-H., Chai T.-T. (2020). Identification of putative cell-entry-inhibitory peptides against SARS-CoV-2 from edible insects: An in silico study. eFood.

[44] Matemu A., Nakamura S., Katayama S. (2021). Health benefits of antioxidative peptides derived from legume proteins with a high amino acid score. Antioxidants.

[45] Forman H.J., Zhang H. (2021). Targeting oxidative stress in disease: Promise and limitations of antioxidant therapy. Nat. Rev. Drug Discov..

[46] Wong F.-C., Xiao J., Wang S., Ee K.-Y., Chai T.-T. (2020). Advances on the antioxidant peptides from edible plant sources. Trends Food Sci. Technol..

[47] Zielińska E., Karaś M., Baraniak B., Jakubczyk A. (2020). Evaluation of ACE, α-glucosidase, and lipase inhibitory activities of peptides obtained by in vitro digestion of selected species of edible insects. Eur. Food Res. Technol..

[48] Zielińska E., Baraniak B., Karaś M. (2018). Identification of antioxidant and anti-inflammatory peptides obtained by simulated gastrointestinal digestion of three edible insects species (*Gryllodes sigillatus*, *Tenebrio molitor*, *Schistocerca gragaria*). Int. J. Food Sci. Technol..

[49] Mudd N., Martin-Gonzalez F.S., Ferruzzi M., Liceaga A.M. (2022). In vivo antioxidant effect of edible cricket (*Gryllodes sigillatus*) peptides using a *Caenorhabditis elegans* model. Food Hydrocoll. Health.

[50] Amada44 Schistocerca gregaria—0061.jpg (Creative Commons Attribution-Share Alike 3.0 Unported license). https://commons.wikimedia.org/wiki/File:Schistocerca_gregaria_-_0061.jpg.

[51] Woo B. *Gryllodes sigillatus* (Permission Granted by Image Owner via Email). https://theincorrigibleentomologist.files.wordpress.com/2018/03/img_7249.jpg?w=768.

[52] Li Z., Zhao S., Xin X., Zhang B., Thomas A., Charles A., Lee K.S., Jin B.R., Gui Z. (2019). Purification and characterization of a novel immunomodulatory hexapeptide from alcalase hydrolysate of ultramicro-pretreated silkworm (*Bombyx mori*) pupa protein. J. Asia-Pac. Entomol..

[53] Vercruysse L., Van Camp J., Morel N., Rougé P., Herregods G., Smagghe G. (2010). Ala-Val-Phe and Val-Phe: ACE inhibitory peptides derived from insect protein with antihypertensive activity in spontaneously hypertensive rats. Peptides.

[54] Hall F., Reddivari L., Liceaga A.M. (2020). Identification and characterization of edible cricket peptides on hypertensive and glycemic in vitro inhibition and their anti-inflammatory activity on RAW 264.7 macrophage cells. Nutrients.

[55] Chen F., Jiang H., Lu Y., Chen W., Huang G. (2019). Identification and in silico analysis of antithrombotic peptides from the enzymatic hydrolysates of *Tenebrio molitor* larvae. Eur. Food Res. Technol..

[56] Montiel-Aguilar L.J., Torres-Castillo J.A., Rodríguez-Servin R., López-Flores A.B., Aguirre-Arzola V.E., Méndez-Zamora G., Sinagawa-García S.R. (2020). Nutraceutical effects of bioactive peptides obtained from *Pterophylla beltrani* (Bolivar & Bolivar) protein isolates. J. Asia-Pac. Entomol..

[57] Mendoza-Salazar A., Santiago-López L., Torres-Llanez M.J., Hernández-Mendoza A., Vallejo-Cordoba B., Liceaga A.M., González-Córdova A.F. (2021). In vitro antioxidant and antihypertensive activity of edible insects flours (mealworm and grasshopper) fermented with *Lactococcus lactis* strains. Fermentation.

[58] (2021). Hypertension.

[59] Aluko R.E. (2015). Antihypertensive peptides from food proteins. Annu. Rev. Food Sci. Technol..

[60] Benigni A., Cassis P., Remuzzi G. (2010). Angiotensin II revisited: New roles in inflammation, immunology and aging. EMBO Mol. Med..

[61] Li G.-H., Le G.-W., Shi Y.-H., Shrestha S. (2004). Angiotensin I–converting enzyme inhibitory peptides derived from food proteins and their physiological and pharmacological effects. Nutr. Res..

[62] Cheung B.M.Y., Li C. (2012). Diabetes and hypertension: Is there a common metabolic pathway?. Curr. Atheroscler. Rep..

[63] Wang R., Zhao H., Pan X., Orfila C., Lu W., Ma Y. (2019). Preparation of bioactive peptides with antidiabetic, antihypertensive, and antioxidant activities and identification of α-glucosidase inhibitory peptides from soy protein. Food Sci. Nutr..

[64] Ahrén B. (2019). DPP-4 inhibition and the path to clinical proof. Front. Endocrinol..

[65] Liu T.-T., Liu X.-T., Chen Q.-X., Shi Y. (2020). Lipase Inhibitors for Obesity: A Review. Biomed. Pharmacother..

[66] ElHeineken Allomyrina dichotoma L3 Larva.JPG (Creative Commons Attribution 3.0 Unported license). https://commons.wikimedia.org/wiki/File:Allomyrina_dichotoma_L3_Larva.JPG.

[67] Xiong Y., Chen Z.H., Zhang F.L., Yu Z.Y., Liu B., Zhang C., Zhao L.N. (2021). A specific selenium-chelating peptide isolated from the protein hydrolysate of *Grifola frondosa*. RSC Adv..

[68] Sonklin C., Alashi A.M., Laohakunjit N., Aluko R.E. (2021). Functional characterization of mung bean meal protein-derived antioxidant peptides. Molecules.

[69] Hall F., Johnson P.E., Liceaga A. (2018). Effect of enzymatic hydrolysis on bioactive properties and allergenicity of cricket (*Gryllodes sigillatus*) protein. Food Chem..

[70] Mnolf Tenebrio molitor Larvae (GFDL & CC ShareAlike 2.0 License). https://commons.wikimedia.org/wiki/File:Tenebrio_molitor_larvae.jpg.

[71] Józefiak A., Engberg R.M. (2017). Insect proteins as a potential source of antimicrobial peptides in livestock production. A review. J. Anim. Feed. Sci..

[72] Veldkamp T., Dong L., Paul A., Govers C. (2022). Bioactive properties of insect products for monogastric animals—A review. J. Insects Food Feed..

[73] Food and Agriculture Organization (2016). The FAO Action Plan on Antimicrobial Resistance 2016–2020.

[74] Llor C., Bjerrum L. (2014). Antimicrobial resistance: Risk associated with antibiotic overuse and initiatives to reduce the problem. Ther. Adv. Drug Saf..

[75] Van Boeckel T.P., Brower C., Gilbert M., Grenfell B.T., Levin S.A., Robinson T.P., Teillant A., Laxminarayan R. (2015). Global trends in antimicrobial use in food animals. Proc. Natl. Acad. Sci. USA.

[76] WHO, FAO, OIE (2021). Antimicrobial Resistance and the United Nations Sustainable Development Cooperation Framework: Guidance for United Nations Country Teams.

[77] Józefiak D., Kierończyk B., Juśkiewicz J., Zduńczyk Z., Rawski M., Długosz J., Sip A., Højberg O. (2013). Dietary nisin modulates the gastrointestinal microbial ecology and enhances growth performance of the broiler chickens. PLoS ONE.

[78] Kierończyk B., Pruszyńska-Oszmałek E., Światkiewicz S., Rawski M., Długosz J., Engberg R.M., Józefiak D. (2016). The nisin improves broiler chicken growth performance and interacts with salinomycin in terms of gastrointestinal tract microbiota composition. J. Anim. Feed. Sci..

[79] Tang Z., Yin Y., Zhang Y., Huang R., Sun Z., Li T., Chu W., Kong X., Li L., Geng M. (2009). Effects of dietary supplementation with an expressed fusion peptide bovine lactoferricin-lactoferrampin on performance, immune function and intestinal mucosal morphology in piglets weaned at age 21 d. Br. J. Nutr..

[80] Yoon J.H., Ingale S.L., Kim J.S., Kim K.H., Lee S.H., Park Y.K., Lee S.C., Kwon I.K., Chae B.J. (2014). Effects of dietary supplementation of synthetic antimicrobial peptide-A3 and P5 on growth performance, apparent total tract digestibility of nutrients, fecal and intestinal microflora and intestinal morphology in weanling pigs. Livest. Sci..

[81] Mohideen H.S., Louis H.P. (2021). Insect antimicrobial peptides—Therapeutic and agriculture perspective. J. Appl. Biotechnol. Rep..

[82] Yi H.Y., Chowdhury M., Huang Y.D., Yu X.Q. (2014). Insect antimicrobial peptides and their applications. Appl. Microbiol. Biotechnol..

[83] Hultmark D., Steiner H., Rasmuson T., Boman H.G. (1980). Insect immunity. Purification and properties of three inducible bactericidal proteins from hemolymph of immunized pupae of *Hyalophora cecropia*. Eur. J. Biochem..

[84] Wu S., Zhang F., Huang Z., Liu H., Xie C., Zhang J., Thacker P.A., Qiao S. (2012). Effects of the antimicrobial peptide cecropin AD on performance and intestinal health in weaned piglets challenged with Escherichia coli. Peptides.

[85] Dai J., Ou W., Yu G., Ai Q., Zhang W., Mai K., Zhang Y. (2020). The antimicrobial peptide cecropin ad supplement alleviated soybean meal-induced intestinal inflammation, barrier damage, and microbial dysbiosis in juvenile turbot, *Scophthalmus maximus*. Front. Mar. Sci..

[86] Wen L.F., He J.G. (2012). Dose-response effects of an antimicrobial peptide, a cecropin hybrid, on growth performance, nutrient utilisation, bacterial counts in the digesta and intestinal morphology in broilers. Br. J. Nutr..

[87] Duclohier H. (2002). How do channel- and pore-forming helical peptides interact with lipid membranes and how does this account for their antimicrobial activity?. Mini Rev. Med. Chem..

[88] Nicolas P. (2009). Multifunctional host defense peptides: Intracellular-targeting antimicrobial peptides. FEBS J..

[89] Wang B., Xie N., Li B. (2019). Influence of peptide characteristics on their stability, intestinal transport, and in vitro bioavailability: A review. J. Food Biochem..

[90] Sánchez-Muros M.J., Barroso F.G., Manzano-Agugliaro F. (2014). Insect meal as renewable source of food for animal feeding: A review. J. Clean. Prod..

[91] Erickson M.C., Islam M., Sheppard C., Liao J., Doyle M.P. (2004). Reduction of Escherichia coli O157:H7 and Salmonella enterica serovar enteritidis in chicken manure by larvae of the black soldier fly. J. Food Prot..

[92] Park S.I., Chang B.S., Yoe S.M. (2014). Detection of antimicrobial substances from larvae of the black soldier fly, *Hermetia illucens* (Diptera: Stratiomyidae). Entomol. Res..

[93] Park S.I., Kim J.W., Yoe S.M. (2015). Purification and characterization of a novel antibacterial peptide from black soldier fly (*Hermetia illucens*) larvae. Dev. Comp. Immunol..

[94] Zhang J., Li J., Peng Y., Gao X., Song Q., Zhang H., Elhag O., Cai M., Zheng L., Yu Z. (2022). Structural and functional characterizations and heterogenous expression of the antimicrobial peptides, Hidefensins, from black soldier fly, *Hermetia illucens* (L.). Protein Expr. Purif..

[95] Cociancich S., Ghazi A., Hetru C., Hoffmann J.A., Letellier L. (1993). Insect defensin, an inducible antibacterial peptide, forms voltage-dependent channels in *Micrococcus luteus*. J. Biol. Chem..

[96] Chernysh S., Gordya N., Suborova T. (2015). Insect antimicrobial peptide complexes prevent resistance development in bacteria. PLoS ONE.

[97] Mouithys-Mickalad A., Schmitt E., Dalim M., Franck T., Tome N.M., van Spankeren M., Serteyn D., Paul A. (2020). Black soldier fly (*Hermetia illucens*) larvae protein derivatives: Potential to promote animal health. Animals.

[98] Li S., Ji H., Zhang B., Zhou J., Yu H. (2017). Defatted black soldier fly (*Hermetia illucens*) larvae meal in diets for juvenile Jian carp (*Cyprinus carpio* var. Jian): Growth performance, antioxidant enzyme activities, digestive enzyme activities, intestine and hepatopancreas histological structure. Aquaculture.

[99] Chu X., Li M., Wang G., Wang K., Shang R., Wang Z., Li L. (2020). Evaluation of the low inclusion of full-fatted *Hermetia illucens* larvae meal for layer chickens: Growth performance, nutrient digestibility, and gut health. Front. Vet. Sci..

[100] Savary S., Willocquet L., Pethybridge S.J., Esker P., McRoberts N., Nelson A. (2019). The global burden of pathogens and pests on major food crops. Nat. Ecol. Evol..

[101] Ekengren S., Hultmark D. (1999). Drosophila cecropin as an antifungal agent. Insect Biochem. Mol. Biol..

[102] Jansen C., Kogel K.-H., Vilcinskas A. (2011). Insect antimicrobial peptides as new weapons against plant pathogens. Insect Biotechnology.

[103] Casteels P., Ampe C., Jacobs F., Vaeck M., Tempst P. (1989). Apidaecins: Antibacterial peptides from honeybees. EMBO J..

[104] Jan P.S., Huang H.Y., Chen H.M. (2010). Expression of a synthesized gene encoding cationic peptide cecropin B in transgenic tomato plants protects against bacterial diseases. Appl. Environ. Microbiol..

[105] Yevtushenko D.P., Romero R., Forward B.S., Hancock R.E., Kay W.W., Misra S. (2005). Pathogen-induced expression of a cecropin A-melittin antimicrobial peptide gene confers antifungal resistance in transgenic tobacco. J. Exp. Bot..

[106] Khademi M., Varasteh-Shams M., Nazarian-Firouzabadi F., Ismaili A. (2020). New recombinant antimicrobial peptides confer resistance to fungal pathogens in tobacco plants. Front. Plant Sci..

[107] Gäde G., Goldsworthy G.J. (2003). Insect peptide hormones: A selective review of their physiology and potential application for pest control. Pest Manag. Sci..

[108] Audsley N., Weaver R.J., Edwards J.P. (2001). In vivo effects of *Manduca sexta* allatostatin and allatotropin on larvae of the tomato moth, *Lacanobia oleracea*. Physiol. Entomol..

[109] Bendena W.G., Donly B.C., Tobe S.S. (1999). Allatostatins: A growing family of neuropeptides with structural and functional diversity. Ann. N. Y. Acad. Sci..

[110] Chong Y., Hayes J.L., Sollod B., Wen S., Wilson D.T., Hains P.G., Hodgson W.C., Broady K.W., King G.F., Nicholson G.M. (2007). The ω-atracotoxins: Selective blockers of insect M-LVA and HVA calcium channels. Biochem. Pharmacol..

[111] Fletcher J.I., Smith R., O’Donoghue S.I., Nilges M., Connor M., Howden M.E.H., Christie M.J., King G.F. (1997). The structure of a novel insecticidal neurotoxin, ω-atracotoxin-HV1, from the venom of an Australian funnel web spider. Nat. Struct. Biol..

[112] Khan S.A., Zafar Y., Briddon R.W., Malik K.A., Mukhtar Z. (2006). Spider venom toxin protects plants from insect attack. Transgenic Res..

[113] Cifuentes-Pagano E.M., Meijles N.D., Pagano J.P. (2015). NOX inhibitors & therapies: Rational design of peptidic and small molecule inhibitors. Curr. Pharm. Des..

[114] Choi S., Colla G., Cardarelli M., Kim H.-J. (2022). Effects of plant-derived protein hydrolysates on yield, quality, and nitrogen use efficiency of greenhouse grown lettuce and tomato. Agronomy.

[115] Colla G., Hoagland L., Ruzzi M., Cardarelli M., Bonini P., Canaguier R., Rouphael Y. (2017). Biostimulant action of protein hydrolysates: Unraveling their effects on plant physiology and microbiome. Front. Plant Sci..

